# ﻿Integrative phylogenomic and morphological evidence for Pseudothelogorgiidae fam. nov., with a redescription of *Pseudothelogorgia hartogi* (Octocorallia, Malacalcyonacea)

**DOI:** 10.3897/zookeys.1261.171874

**Published:** 2025-11-28

**Authors:** Kaveh Samimi-Namin, Catherine S. McFadden

**Affiliations:** 1 Marine Evolution and Ecology Group, Naturalis Biodiversity Center, P.O. Box 9517, 2300 RA Leiden, Netherlands Naturalis Biodiversity Center Leiden Netherlands; 2 Department of Biology, University of Oxford, Oxfordshire, Oxford OX1 3SZ, UK University of Oxford Oxford United Kingdom; 3 Natural History Museum, Cromwell Road, London SW7 5BD, UK Natural History Museum London United Kingdom; 4 Department of Biology, Harvey Mudd College, Claremont, CA 91711, USA Harvey Mudd College Claremont United States of America

**Keywords:** Arabian Sea, deep-water corals, Indian Ocean, mesophotic, new family, Oman, systematics, taxonomy, ultraconserved elements (UCEs)

## Abstract

The octocoral genus *Pseudothelogorgia* van Ofwegen, 1994 has presented taxonomic challenges since its original description, with its higher-level placement uncertain and obscured by morphological convergence and lack of molecular data. Here, we integrate detailed morphological examination with phylogenomic analyses of newly collected shallow-water specimens from the Gulf of Oman, alongside a redescription of the type material. Our results recover *Pseudothelogorgia* as a deeply divergent lineage within Malacalcyonacea, sister to Plexauridae but unrelated to Keroeididae, the family in which it was formerly placed. Distinct morphological characters, together with strong phylogenomic support, warrant the establishment of Pseudothelogorgiidae**fam. nov.** The shallow-water occurrences documented here extend the known depth range of the genus from mesophotic to shallow reef habitats, indicating ecological plasticity and highlighting the need for targeted exploration of under-surveyed Indian Ocean coral assemblages.

## ﻿Introduction

Resolving evolutionary relationships in octocorals is often hampered by morphological convergence, which has long complicated the systematics of deep-water gorgonian octocorals and obscured true relationships among genera (Bayer 1991; McFadden et al. 2022). The genus *Pseudothelogorgia* van Ofwegen, 1994, the focus of the present study, exemplifies this challenge, having undergone multiple reclassifications (van Ofwegen 1990, 1994) and remaining phylogenetically unresolved for more than three decades.

The history of *Pseudothelogorgia* begins in the Arabian Sea, from where van Ofwegen (1990) described *Lignella hartogi* based on material collected during the Anton Bruun Expedition (1963–1964). The species was placed in *Lignella* because of its morphological resemblance to *L. richardii* (Lamouroux, 1816) from the western Atlantic (see Bayer 1961, 1991). However, the identity of *L. richardii* has long been uncertain. After re-examining available material and historical descriptions, Bayer (1991) concluded that the true identity of *Gorgonia richardii* Lamouroux, 1816—and by extension the definition of *Lignella*—could not be resolved without study of the type specimens, which he considered lost at the time given the ambiguities in the original description, but these specimens are now known to exist (pers. obs.).

To address this taxonomic ambiguity, Bayer (1991) established the genus *Thelogorgia* within Keroeididae for a group of western Atlantic species previously assigned to *Lignella richardii*. This new genus was based on well-documented Atlantic material and distinguished from the Indo-Pacific genus *Keroeides*, which, despite some morphological similarities, represents a separate lineage. Notably, *Lignella hartogi* from the Indian Ocean was not re-examined in Bayer’s (1991) revision, leaving its generic and family placement unresolved.

Acknowledging these uncertainties, van Ofwegen (1994) compared *L. hartogi* with members of *Thelogorgia*, showing that *L. hartogi* was morphologically distinct—particularly in sclerite structure—from its Atlantic counterparts. He therefore established *Pseudothelogorgia* for this Indian Ocean lineage, separating it from both the problematic Atlantic “*Lignella*” group and from *Keroeides*. Subsequent identifications of *Pseudothelogorgia* from the Philippines (68 m depth; by F.M. Bayer, USNM) and Palau (207 m depth; by S.D. Cairns, USNM) extended its known range. The genus was not encountered again until its rediscovery and first *in situ* photographs in shallow water (18 m depth) at the Daymaniyat Islands, Gulf of Oman (Samimi-Namin et al. 2011). Additional collections along the Oman coastline have since enabled detailed morphological and molecular analyses.

Despite these advances, the higher-level placement of *Pseudothelogorgia* has remained unclear. Traditional classifications placed the genus in Keroeididae—a family defined largely by morphological similarity of the axis—but such morphological similarity alone does not establish a natural (i.e. evolutionarily coherent) group, and their relationships remain unclear in the absence of molecular data (van Ofwegen 1990, 1994; Bayer 1991). By establishing *Pseudothelogorgia*, van Ofwegen (1994) suggested that earlier family concepts, particularly Keroeididae, did not reflect natural lineages and instead acted as a catch-all for superficially similar but unrelated taxa. As Bayer (1991) noted, species and higher taxa in this group were often delineated through subjective interpretation of variable morphological traits, frequently from limited or poorly preserved material. He emphasized the need for “larger suites of specimens and, crucially, biochemical and other non-morphological data” to resolve these ambiguities, a need now addressed by molecular approaches.

Recent collections from the Gulf of Oman, combined with molecular phylogenetic analyses, resolve some of these long-standing issues. Our results show that *Pseudothelogorgia* is a deeply divergent, isolated lineage within Malacalcyonacea, not closely related to any currently recognized family, including Keroeididae. The recent phylogenomic study of Quattrini et al. (2024), which included the same *Pseudothelogorgia* voucher examined here, independently recovered the genus as a distinct lineage and recommended family-level recognition, pending confirmation of species identity, which we provide here. Our study confirms that identity, documents its distinctive morphology, and supports the establishment of Pseudothelogorgiidae fam. nov. while clarifying its position within Malacalcyonacea.

## ﻿Material and methods

Field observations and specimen collection have been conducted in the Gulf of Oman and the Arabian Sea since 2008 as part of ongoing biodiversity surveys in the northwestern Indian Ocean. *In situ* photographs were taken using a compact underwater camera, and depths were recorded with a dive computer. Specimens were carefully detached, photographed, and preserved in 70% ethanol, with tissues retained for molecular analysis in 96% ethanol. Voucher specimens used for morphological studies are deposited in the RMNH, UF, USNM, and NHMW collections; additional material used for the molecular analyses is deposited in the institutions listed in the abbreviations and in Suppl. material 1.

### ﻿Abbreviations

**CAS** California Academy of Sciences, San Francisco, United States

**MZUCR** Museo de Zoología, Universidad de Costa Rica, San José, Costa Rica

**NHMW** Naturhistorisches Museum Wien, Vienna, Austria

**NIWA** National Institute of Water and Atmospheric Research, Wellington, New Zealand

**RMNH** Naturalis Biodiversity Center (formerly Rijksmuseum van Natuurlijke Historie, RMNH), Leiden, The Netherlands

**SBMNH** Santa Barbara Museum of Natural History, Santa Barbara, United States

**UF** Florida Museum of Natural History, University of Florida, Gainesville, United States

**USNM** Smithsonian National Museum of Natural History, Washington DC, United States

### ﻿Morphological studies

To identify the material, sclerites were extracted by dissolving the tissues in 10% sodium hypochlorite, followed by thorough rinsing in fresh water. For scanning electron microscopy (SEM), sclerites were rinsed with double-distilled water, air-dried at room temperature, mounted on aluminium stubs with double-sided carbon tape, coated with Platinum-Palladium (Pt/Pd), and examined under JEOL JSM-IT510 at 10 kV. All specimens, microscope slides, and stubs are deposited in the Cnidaria collection (RMNH.COEL.) of Naturalis Biodiversity Center, Leiden, the Netherlands.

### ﻿Molecular and phylogenetic analyses

DNA was isolated from the ethanol-preserved tissue sample, UF 16054, using a Qiagen DNeasy Blood & Tissue kit and manufacturer’s protocol. DNA was quantified using a Qubit 2.0 fluorometer and quality-checked for 260:280 and 260:230 ratios with a NanoDrop spectrophotometer. 1000 ng of DNA was sent to Arbor Biosystems (Ann Arbor, MI) for library preparation, target-enrichment and sequencing. Libraries were prepared using a Kapa Hyper Prep Kit (Kapa Biosystems) with dual-indexed iTru adaptors, and myBaits protocol v. 4 (Arbor Biosystems) was used to target and enrich pools of libraries using the octocoral-v2 bait set of Erickson et al. (2021). Enriched libraries were sequenced on one lane of Illumina HiSeq 2500 (150 bp PE reads).

**Figure 1.**
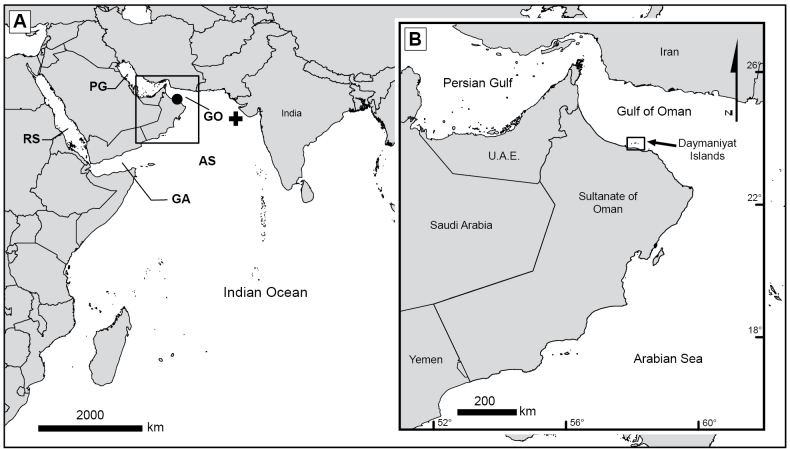
**A.** Regional map showing the localities of studied material of *Pseudothelogorgia hartogi*. RS = Red Sea; PG = Persian Gulf; GA = Gulf of Aden; GO = Gulf of Oman; AS = Arabian Sea. The cross marks the type locality (Arabian Sea), and the circle indicates additional material from Oman; **B.** Enlarged view of the Gulf of Oman showing the Daymaniyat Islands collection site.

Sequence reads were processed as described by Quattrini et al. (2024). Briefly, reads were trimmed of adaptors and low-quality bases using Trimmomatic v. 0.39 (Bolger et al. 2014) and assembled into contigs using SPAdes v. 3.13.0 (Bankevich et al. 2012). PHYLUCE v. 1.7 (Faircloth 2016) was used to match contigs to UCEs and exons and extract fasta files. Fastas for UF 16054 were then aligned with those of octocorals belonging to families that were phylogenetically close to that sample in the analyses of Quattrini et al. (2024). These included representatives of all available genera in families Plexauridae and Pterogorgiidae, with Isididae, Astrogorgiidae, Discophytidae, Keroeididae, and Taiaroidae as outgroup taxa (Suppl. material 1). Sequences were aligned with MAFFT v. 7.130b (Katoh and Standley 2013) and concatenated alignments with 65% or 75% taxon occupancy were used to construct maximum likelihood trees with IQTree v. 2.3.1 (Minh et al. 2020). The best model of nucleotide substitution for each partition was found with ModelFinder (−m MFP, Kalyaanamoorthy et al. 2017) and the analyses were run with 1000 ultrafast bootstraps (−bb 1000, Hoang et al. 2018) and the SH-like approximate likelihood ratio test (−alrt 1000; Guindon et al. 2010) (Fig. 1).

## ﻿Results

### ﻿Molecular results

**Figure 2.**
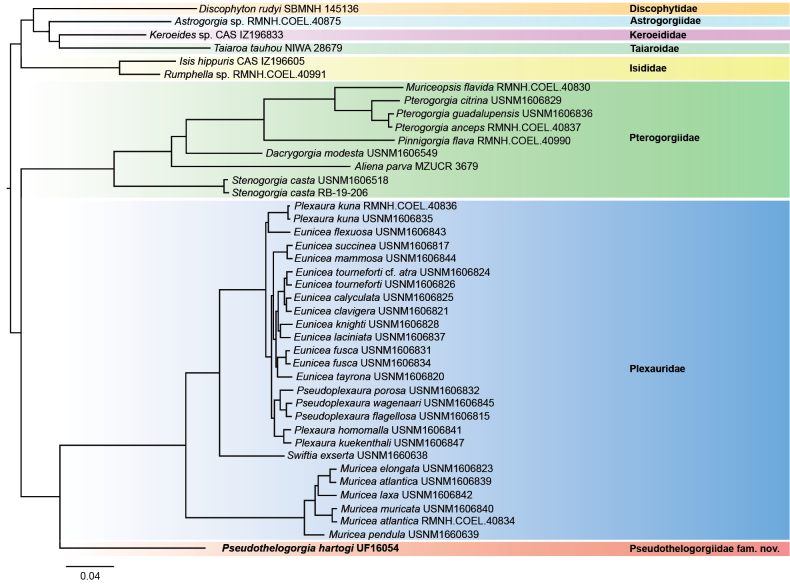
Maximum-likelihood tree based on concatenated alignment (75% taxon occupancy) of 1289 conserved elements (UCEs and exons). All nodes have 100% support from 1000 UF bootstraps and SH-aLRT. The tree includes a subset of samples analysed previously by Quattrini et al. (2024).

A total of 1997 ultraconserved and exon loci were recovered from sample UF 16054, of which 1289 were included in the 75% taxon occupancy matrix analysed here. A matrix with 65% occupancy (2034 loci) yielded comparable results. Both trees recovered 100% support (UF bootstraps and SH-aLRT) for all nodes, with *Pseudothelogorgia* as the sister group to family Plexauridae (Fig. 2). The genus *Keroeides*, with which *Pseudothelogorgia* was previously classified in family Keroeididae, was recovered among the outgroup taxa where it was sister to the soft coral family Taiaroidae. This topology matches that of Quattrini et al. (2024), who included the same sequence data in a taxon-comprehensive analysis of Octocorallia.

### ﻿Systematic account

**Octocorallia Haeckel, 1866**

**Malacalcyonacea McFadden, van Ofwegen & Quattrini, 2022**

#### ﻿ Pseudothelogorgiidae fam. nov.

Taxon classificationAnimaliaMalacalcyonaceaPseudothelogorgiidae

C60E568E-2E2B-5240-ACF7-40B661A195FD

https://zoobank.org/C977A75D-1C10-40DF-A814-9871DBFECDA1

##### Type genus.

*Pseudothelogorgia* van Ofwegen, 1994.

Excluded from Keroeididae Kinoshita, 1910 (see van Ofwegen 1990, 1994).

##### Included genus.

*Pseudothelogorgia* van Ofwegen, 1994.

##### Diagnosis.

Octocorals with a skeletal axis of consolidated sclerites embedded within a proteinaceous or calcitic matrix with hollow central core (no cross-chambering observed). Axial sclerites smooth, fused, densely packed around the central core forming pavement-like surface layer. Colonies arborescent, branching irregularly to dichotomously; branches cylindrical. Polyps monomorphic, non-retractile into the thin coenenchyme, arranged spirally along branches. Longitudinal furrows present along stem and branches. Polyps with tuberculate rods and spindles arranged as points; collaret absent; tentacles with rods; pharyngeal sclerites absent. Sclerites of the coenenchyme are rods and spindles with simple or complex tubercles. Capstans present in coenenchyme of the main stem. Azooxanthellate.

##### Remarks.

The genus *Pseudothelogorgia* and its type species (*P. hartogi*) were previously included in Keroeididae Kinoshita, 1910 (van Ofwegen 1990, 1994). However, recent molecular phylogenetic analyses (this study; Quattrini et al. 2024) and unique morphological features demonstrate that *Pseudothelogorgia* represents a distinct lineage, supporting its exclusion from Keroeididae and the establishment of Pseudothelogorgiidae fam. nov.

Pseudothelogorgiidae fam. nov. differs from Plexauridae in having a thin, translucent stem coenenchyme containing capstans, rods, and spindles with complex tubercles, and an axial cortex of fused sclerites surrounding a hollow core, rather than the thick, loculated coenenchyme with separate inner and outer layers and unfused axial cortex sclerites typical of Plexauridae.

##### Distribution.

Indo-West Pacific, shallow to mesophotic (18–207 m depth).

#### ﻿ Pseudothelogorgia

Taxon classificationAnimaliaMalacalcyonaceaPseudothelogorgiidae

van Ofwegen, 1994

3F6C66CF-8DF8-5F09-9CFE-C309A6A1F2B9

Pseudothelogorgia van Ofwegen, 1994: 19–21, figs 1–2 [original description, type species designated].
— Pseudothelogorgia: Samimi-Namin et al. 2011: fig. 1 [first *in situ* record, Gulf of Oman, 18 m depth].
= Lignella (partim): van Ofwegen 1990: 164–168 [as Lignella hartogi; now Pseudothelogorgia hartogi (van Ofwegen, 1990)].

##### Type species.

*Lignella hartogi* van Ofwegen, 1990, by original designation.

##### Remarks.

*Pseudothelogorgia* was established by van Ofwegen (1994) to accommodate *Lignella hartogi* van Ofwegen, 1990, which was originally assigned to Keroeididae. The genus is distinguished from both Atlantic *Thelogorgia* and Indo-Pacific *Keroeides* by unique sclerite morphology and, as shown here, by molecular divergence.

Two specimens—USNM 49819 (Philippines, 68 m depth) and USNM 1006517 (Palau, 207 m depth)—were identified by F.M. Bayer and S.D. Cairns but have not been examined by us in detail. However, SEM images provided by S.D. Cairns confirm their placement in the genus. These records are currently included in *Pseudothelogorgia*, but given their geographic distance from the type locality, future molecular data and detailed sclerite analyses will be needed to determine whether they represent *P. hartogi* or closely related, distinct species.

##### Distribution.

Indo-Pacific, shallow to mesophotic (18–207 m depth).

#### ﻿ Pseudothelogorgia hartogi

Taxon classificationAnimaliaMalacalcyonaceaPseudothelogorgiidae

(van Ofwegen, 1990)

3BC36559-C6F6-5562-A149-D6003AE46D7B

Figs 3, 4, 5, 6, 7, 8, 9, 10

= Lignella hartogi van Ofwegen, 1990: 164–168, figs 1–3 [original description].
— Pseudothelogorgia hartogi (van Ofwegen, 1990): van Ofwegen, 1994: 19–21, figs 1–2 [new comb.].
— Pseudothelogorgia hartogi: Samimi-Namin et al. 2011: fig. 1 [first live record, Daymaniyat Islands, Gulf of Oman, 18 m depth].

##### Type locality.

Arabian Sea, 22.53°N, 68.12°E, 57 m (holotype USNM 83607).

##### Material examined.

***Holotype***: India • Arabian Sea, India, Gujarat; R/V Anton Bruun; Cruise 4B; Sta. 221A, 22.53°N, 68.12°E; 18 November 1963; sediment type sandy green clay (mud); 57 m depth; USNM 83607.

***Paratypes***: India • 2 colonies and some fragments, Arabian Sea, India, Gujarat; R/V Anton Bruun; Cruise 4B; Sta. 221A; 22.53°N, 68.12°E; 18 November 1963; sediment type sandy green clay (mud); 57 m depth; RMNH.COEL.17782 • 4 colonies and many fragments; same collection data as preceding; USNM 81904 (microscope slides only).

##### Other material.

Oman • (one microscope slide 1602), Gulf of Oman, Daymaniyat Islands (D4); 23.8620°N, 58.1042°E; 18 m depth; rocky wall; coll. K. Samimi-Namin; 23 May 2009; RMNH.COEL.39634 • Gulf of Oman, Daymaniyat Islands; Sta. 59; coll. K. Samimi-Namin; 29 January 2022, 15–17 m depth, UF 16054 (BOMAN 12120).

##### Description.

The present redescription of *Pseudothelogorgia hartogi* is based primarily on paratype material (RMNH.COEL.17782). The holotype (USNM 83607) was examined from fragments and permanent slides housed in the RMNH collection, but the colony material was insufficient for SEM. Paratypes, collected at the same station and date as the holotype (Cruise 4B, Sta. 221A), are morphologically identical in colony form and sclerite composition (van Ofwegen 1990, 1994). Accordingly, SEM images and detailed sclerite descriptions are derived from paratypes, with consistency between holotype fragments, slides, and paratypes confirmed in this study. In addition, recently collected material from the Gulf of Oman (UF 16054, RMNH.COEL.39634) provided supplementary information, including molecular data and the *in situ* photographs of living colonies.

**Figure 3.**
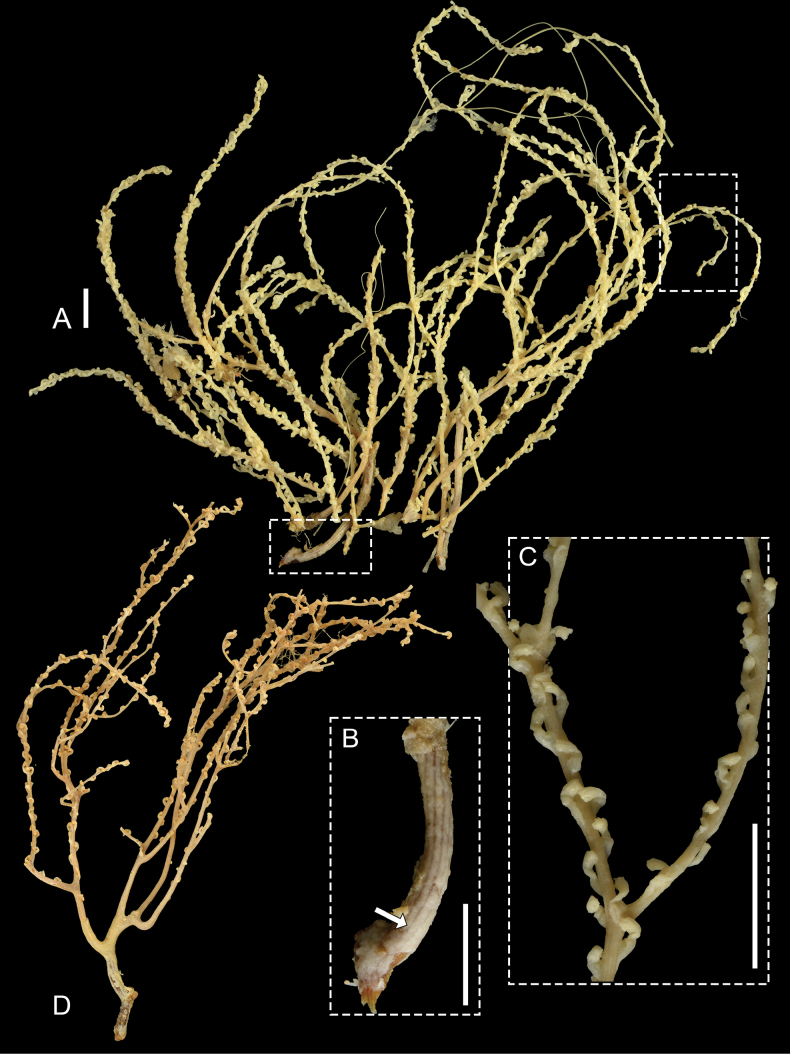
*Pseudothelogorgia hartogi* preserved colonies. **A.** Paratype (RMNH.COEL.17782); **B.** High-magnification view of the main stem showing longitudinal furrows; **C.** Polyp arrangement around the branches; **D.** Additional colony (RMNH.COEL.39634). Scale bars: 1 cm.

The paratypes are arborescent colonies about 15 cm tall with sparse lateral branching (Fig. 3A). The branches arise irregularly but are mostly dichotomous, and reach a length of 7 cm. The polyps are cylindrical, arranged in a spiral around the branches (Fig. 3C). They are not retractable into the coenenchyme of the branches, but they are contracted and curved inward toward the axis. The main stem of the colony lacks polyps. There are several parallel furrows on the main axis which extend all the way up and into the smaller branches (Fig. 3B).

**Figure 4.**
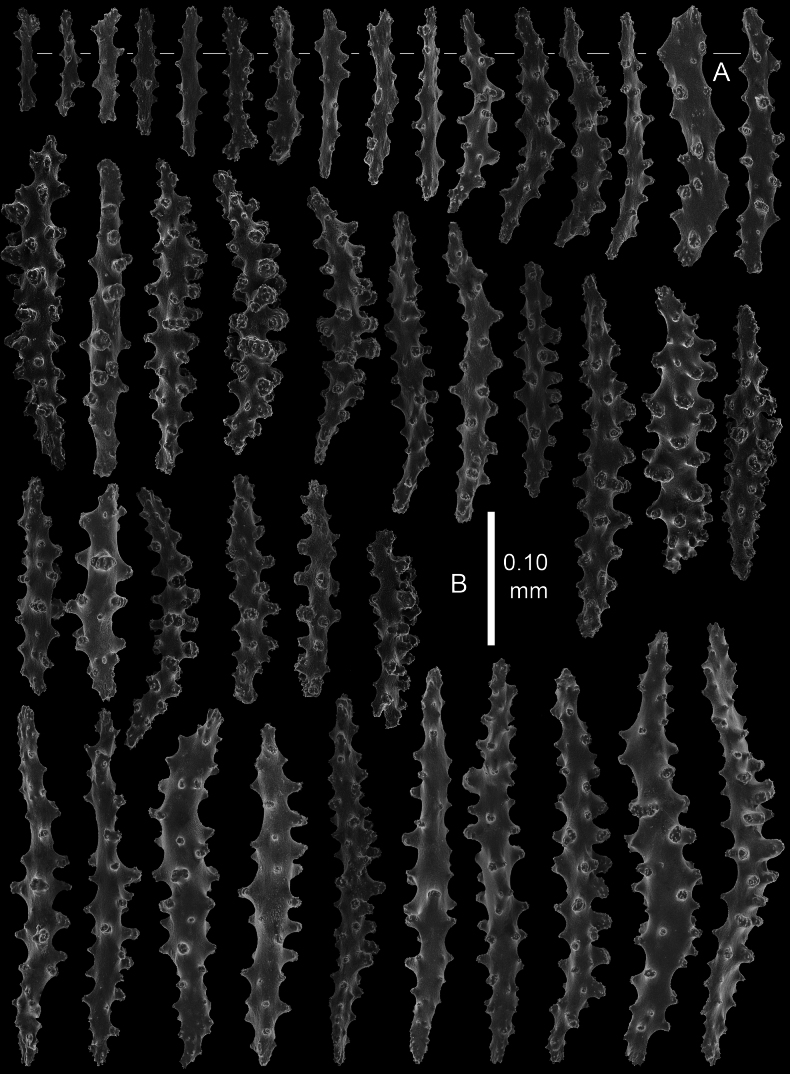
*Pseudothelogorgia hartogi*, paratype RMNH.COEL.17782. **A.** Polyp sclerites; **B.** Coenenchymal sclerites.

Polyps and coenenchyme of branches with slender spindles up to about 0.45 mm long with spines and simple tubercles (Fig. 4).

The polyps have similar spindles up to 0.25 mm long (Fig. 4A), arranged in eight longitudinal tracts when contracted. At the base of the polyps the spindles are irregularly arranged more obliquely and transversely. Tentacles with small spiny rods up to 0.10 mm long (Fig. 4A). The length of the spindles decreases from the main branch and base of the polyp towards the tentacles.

**Figure 5.**
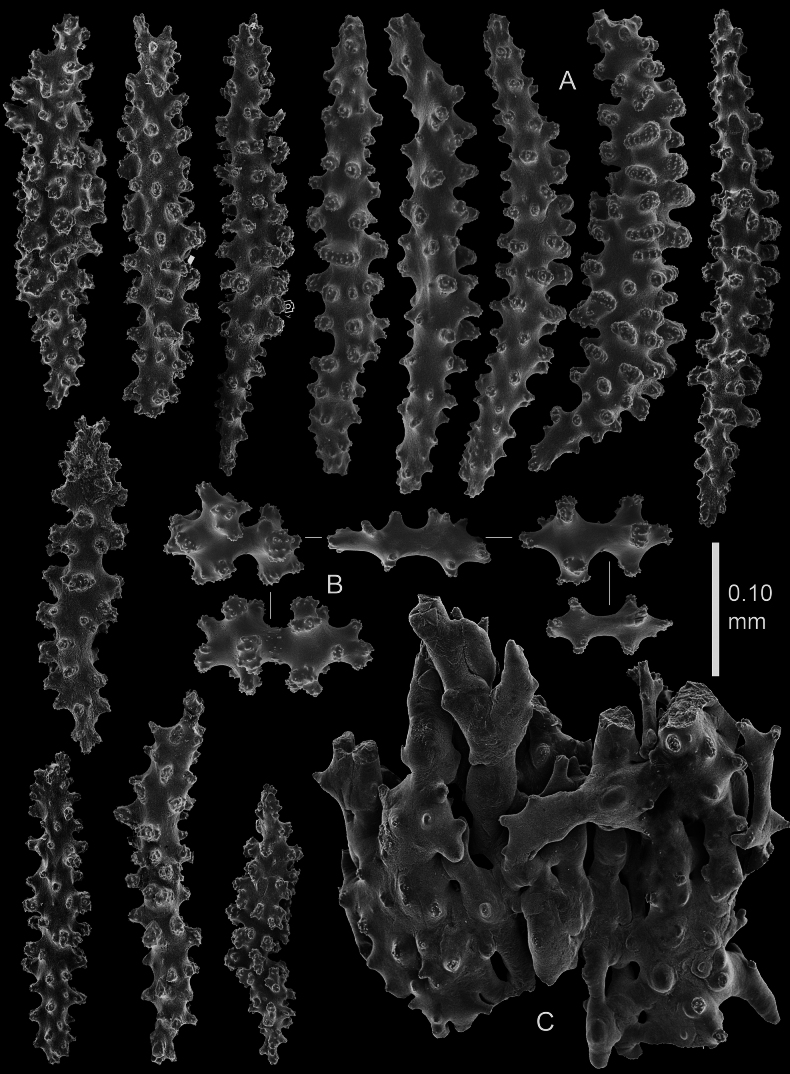
*Pseudothelogorgia hartogi* paratype RMNH.COEL.17782. **A.** Coenenchymal sclerites with complex tubercles; **B.** Coenenchymal capstans of the main stem; **C.** Fused sclerites from the basal part of the coenenchyme.

Coenenchyme of branches is thin and translucent, with spindles with simple and complex tubercles up to 0.45 mm long (Fig. 5A). Some of these large spindles are bent and have larger complex tubercles on one side (Fig. 5A). Capstans present only in the coenenchyme of the main stem (Fig. 5B).

Axial cortex composed of fused sclerites (Fig. 5C) surrounding a central tube.

##### Colour.

**Figure 6.**
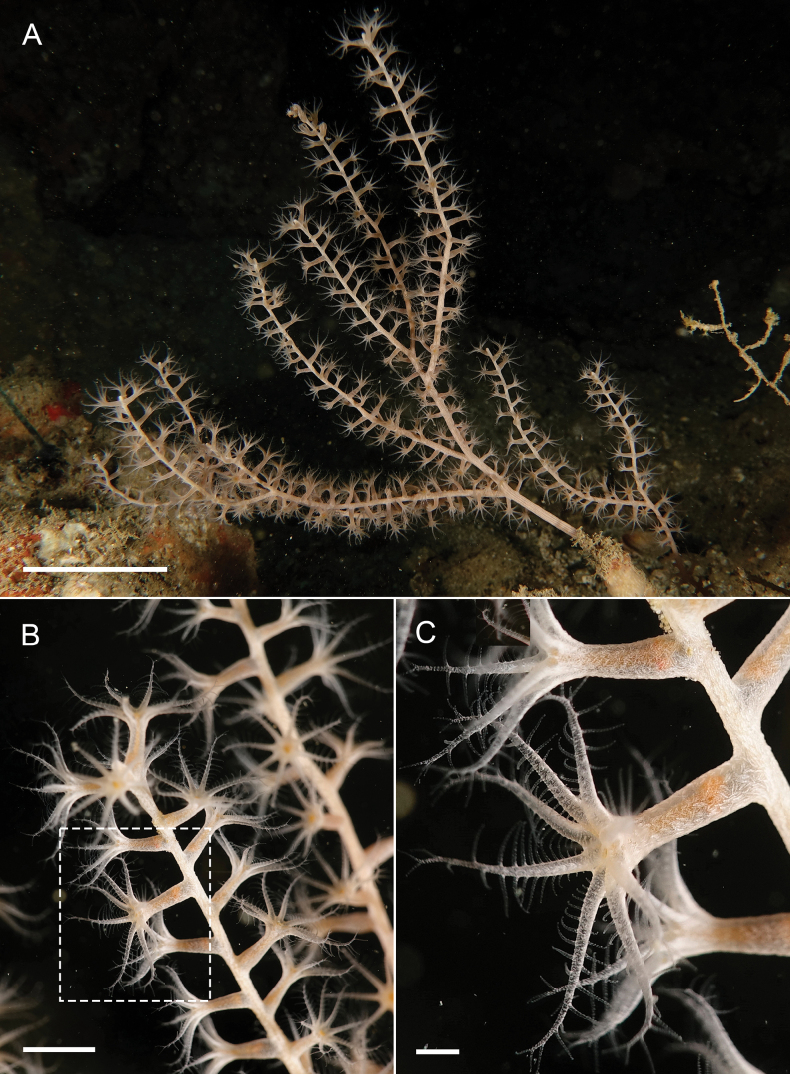
*Pseudothelogorgia hartogi* from Daymaniyat Islands, UF 16054 (BOMAN 12120). **A.** Colony at 20 m depth; **B.** Branches and polyps; **C.** Morphological details of polyps in panel B. Scale bars: 10 cm (**A**); 2 cm (**B**); 3 mm (**C**).

Preserved colonies are cream. Tissue on live colonies (additional material) is translucent, and the colonies appear light cream; polyps translucent (Fig. 6). All sclerites colourless.

##### Variations.

**Figure 7.**
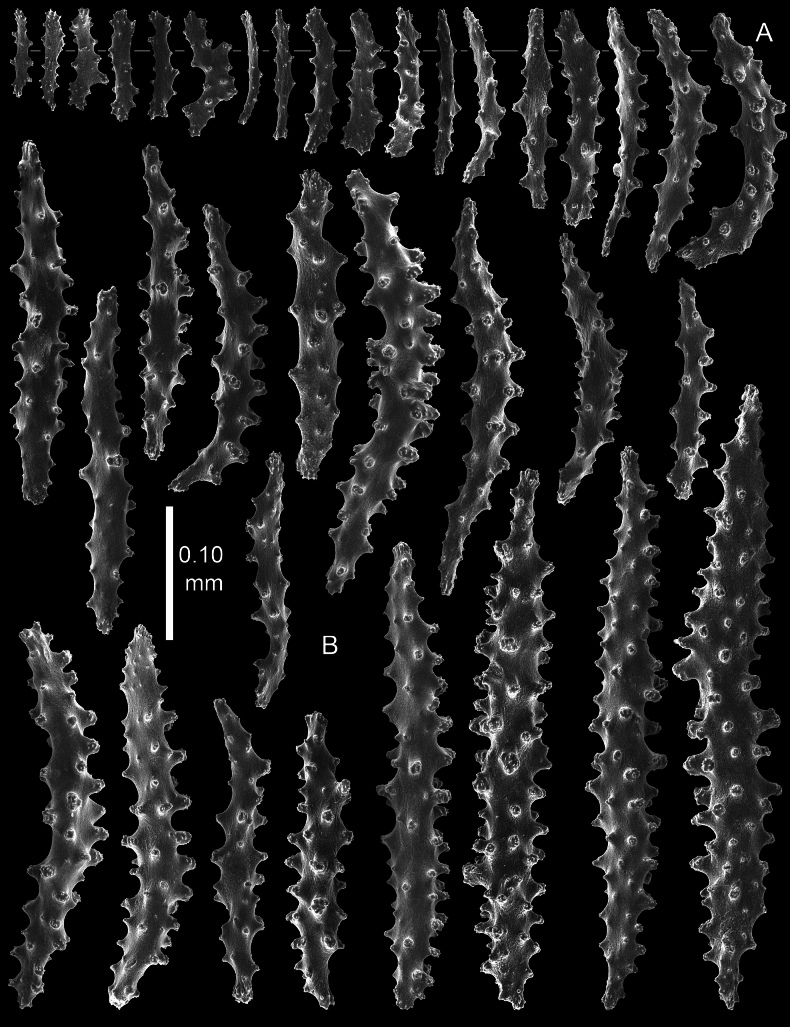
*Pseudothelogorgia hartogi*UF 16054 (BOMAN 12120). **A.** Polyp sclerites; **B.** Coenenchymal sclerites.

**Figure 8.**
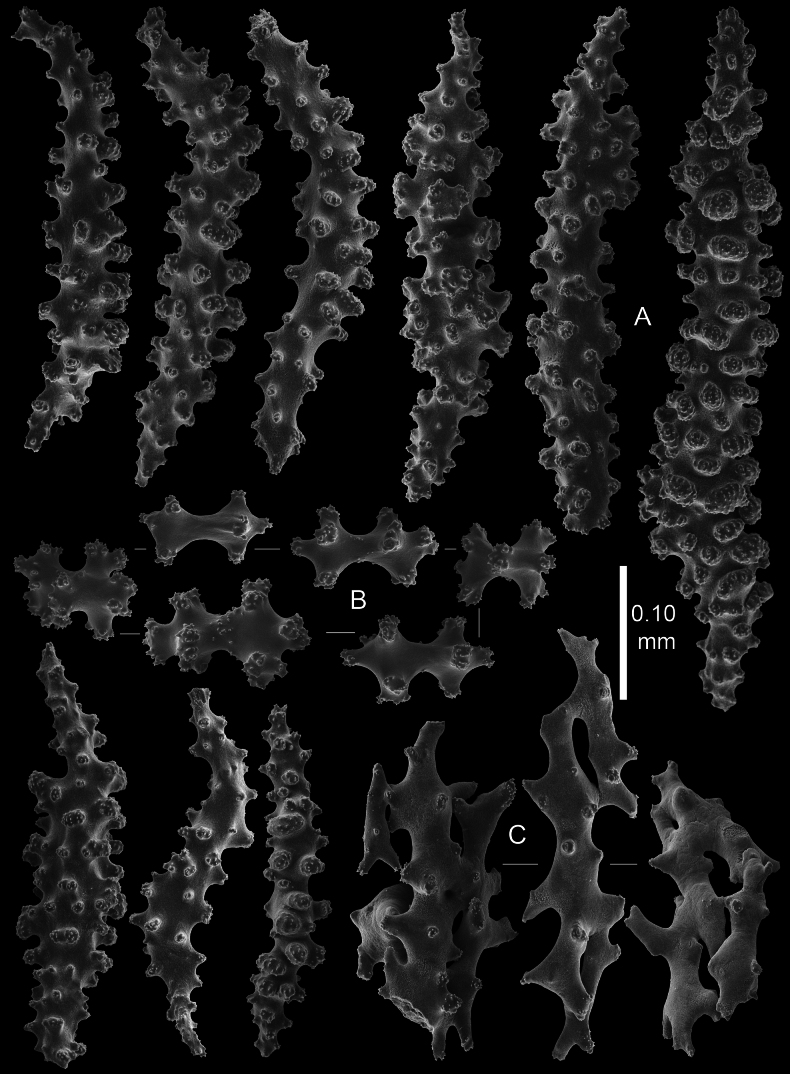
*Pseudothelogorgia hartogi*UF 16054 (BOMAN 12120). **A.** Coenenchymal sclerites with complex tubercles; **B.** Coenenchymal capstans of the main branch; **C.** Fused sclerites from the basal part of the coenenchyme.

**Figure 9.**
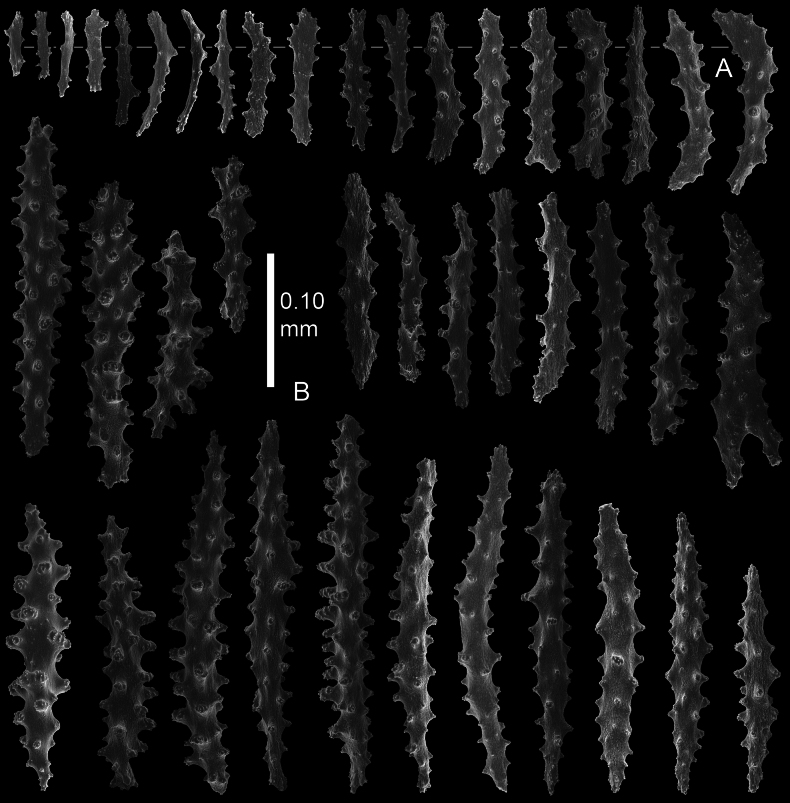
*Pseudothelogorgia hartogi*RMNH.COEL.39634. **A.** Polyp sclerites; **B.** Coenenchymal sclerites with simple tubercles.

**Figure 10.**
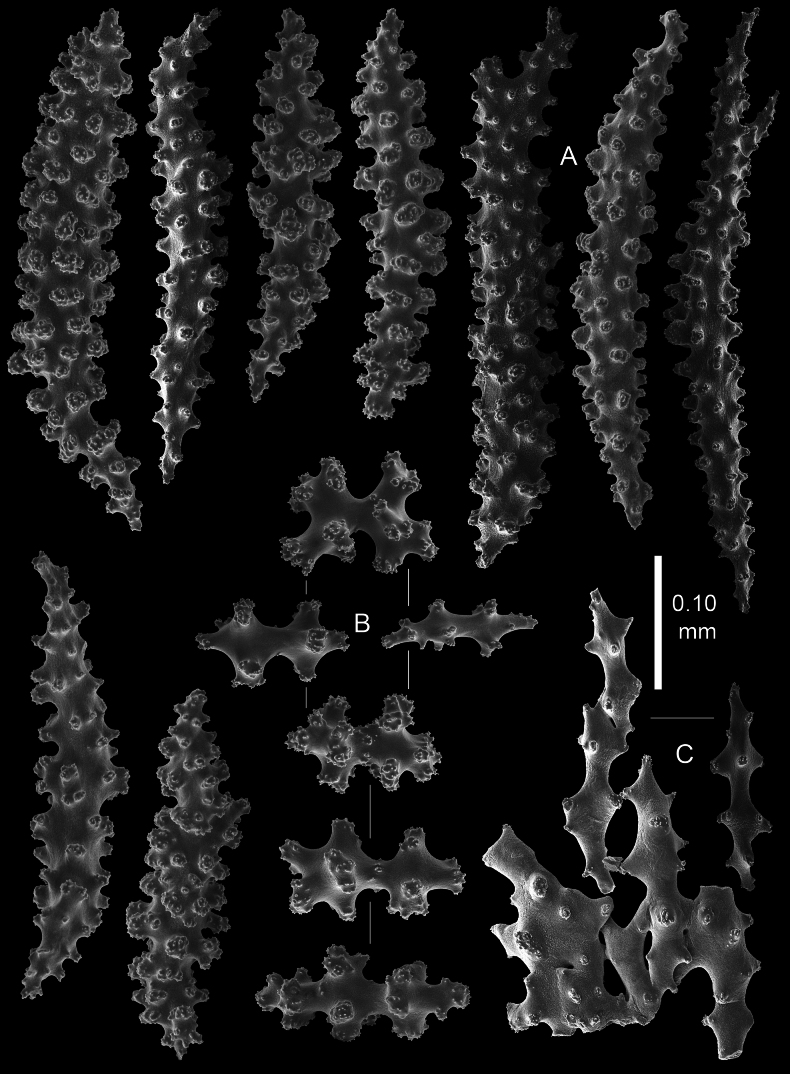
*Pseudothelogorgia hartogi*RMNH.COEL.39634. **A.** Coenenchymal sclerites with complex tubercles; **B.** Coenenchymal capstans of the main branch; **C.** Fused sclerites from the basal part of the coenenchyme.

**Figure 11.**
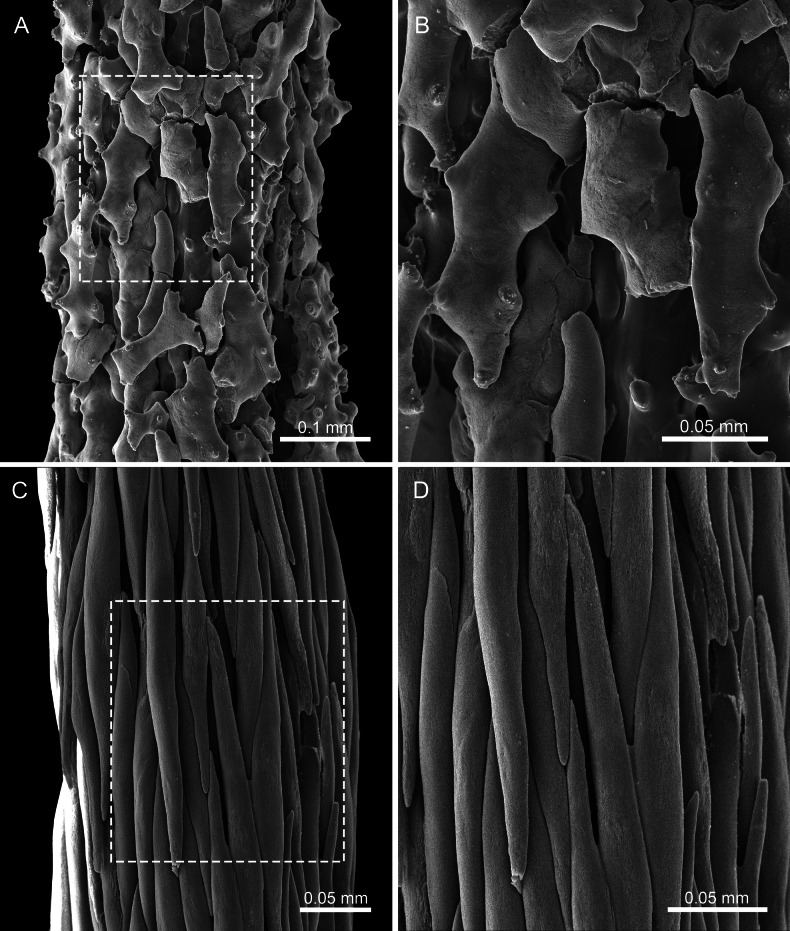
*Pseudothelogorgia hartogi*UF 16054 (BOMAN 12120); **A, B.**SEM of the axis surface at different magnifications; **C, D.** Axis of the type specimen of *Keroeides erythraea* (Kükenthal, 1913), NHMW 2439, Red Sea.

All examined material is consistent in colony morphology and sclerite characters, with very little intraspecific variation observed. Polyps are spirally arranged in all specimens; in preserved colonies they appear contracted and curved inward (Fig. 3C), whereas in living colonies they are extended (Fig. 6). Preserved colonies are cream to beige (Fig. 3), while living colonies are light cream with translucent polyps (Fig. 6). Sclerite types are consistent across specimens, with only minor variation in the relative abundance of capstans and in the development of tubercles (Figs 4, 5, 7–10). The fused, pavement-like axial cortex is uniform in all material (Fig. 11A, B).

##### Remarks.

The sclerites of *Pseudothelogorgia hartogi* differ from species of the genus *Thelogorgia* in the following characters: the fused sclerites of the axial cortex and the anthocodial rods are smaller, and no pharyngeal spindles are present. The sclerites of the distal part of the polyps of *P. hartogi* are distinctly smaller and the coenenchyme of the stem contains spindles, rods and capstans. The material (RMNH.COEL.17194) incorrectly identified by van Ofwegen in 1990 as *Lignella richardii* and used for comparison with *Pseudothelogorgia hartogi* is now identified as *Thelogorgia vossi* Bayer, 1991.

##### Distribution.

Arabian Sea, Gulf of Oman; Indian Ocean.

## ﻿Discussion

The phylogenomic analysis provides compelling evidence that Pseudothelogorgiidae fam. nov. represents a distinct lineage within Malacalcyonacea. *Pseudothelogorgia hartogi* (UF 16054) forms a deeply divergent clade (Fig. 2), placed as sister to Plexauridae. In concert with significant morphological differences, the substantial genetic distance between these groups supports recognition at the family level. This position is consistent with Quattrini et al. (2024), who recovered *Pseudothelogorgia* as a unique lineage and recommended family-level recognition. Our study strengthens that conclusion by confirming species identity and expanding morphological comparisons.

Historically, *Pseudothelogorgia* was assigned to Keroeididae (van Ofwegen 1990, 1994) based on similarities in axial structure and sclerite forms. Following the most recent revision of Malacalcyonacea (McFadden et al. 2022), only *Keroeides* remained in Keroeididae, with *Pseudothelogorgia* and *Thelogorgia* left *incertae sedis* pending molecular evidence. Our results now resolve this uncertainty: *Pseudothelogorgia* is only distantly related to *Keroeides*, underscoring the artificial nature of earlier family concepts based solely on morphology, a problem long recognized in octocoral systematics (Bayer 1991; van Ofwegen 1994). The status of *Thelogorgia* and other former Keroeididae such as *Lignella* remains unresolved pending re-examination of types and molecular data.

Morphologically, Pseudothelogorgiidae fam. nov. differs markedly from Plexauridae. The stem coenenchyme is thin and translucent, lacking loculation and longitudinal canals, and the axial cortex is formed by smooth, fused sclerites surrounding a hollow core that is not cross-chambered. Polyps are non-retractile and spirally arranged. In addition, coenenchymal sclerites are simple capstans, rods, and spindles rather than the robust clubs and leaf-clubs typical of some Plexauridae.

Pseudothelogorgiidae fam. nov. is distinguished from Keroeididae by its fused axial cortex sclerites, distinctive sclerite types, and absence of the prominent calyces and tentacular scales characteristic of *Keroeides*. The axis of *Pseudothelogorgia hartogi* (Fig. 11A, B) is composed of irregular, tuberculate plate-like sclerites that interlock closely to form a compact outer layer, giving the axis surface a distinctly uneven texture. In contrast, the axis of *Keroeides erythraea* (Fig. 11C, D) consists of elongate, rod-like sclerites aligned longitudinally along the axis, producing a markedly fibrous appearance. This fundamental difference in sclerite form and arrangement indicates that despite superficial similarity in overall axis consolidation, *Pseudothelogorgia* and *Keroeides* are structurally distinct. These morphological synapomorphies, together with the molecular evidence, justify recognition of Pseudothelogorgiidae fam. nov. as a separate family.

Ecologically, most records of *Pseudothelogorgia* are from mesophotic and deep-water habitats (>30 m depth). Our collections, however, document *P. hartogi* in shallow reefs (<20 m depth) in the Gulf of Oman. This finding extends the known depth range of the genus, suggesting greater ecological tolerance than previously recognized. Such habitat breadth points to potential ecological plasticity and unrecognized diversity within Indian Ocean octocorals. It also raises questions about dispersal capacity, reproductive strategies, and possible cryptic speciation along depth gradients, highlighting the importance of surveying underexplored shallow habitats.

More broadly, the strong phylogenetic support recovered across all families in our analyses confirms the robustness of the current molecular framework for octocoral systematics (McFadden et al. 2022). While morphology had already suggested placement of *Pseudothelogorgia* within Malacalcyonacea, the phylogenomic results clarify its exact position as a deeply divergent lineage, unrelated to Keroeididae and only distantly allied to Plexauridae. This demonstrates the critical role of genome-scale data in resolving higher-level relationships among morphologically convergent groups.

In conclusion, our study resolves the systematic position of *Pseudothelogorgia* and provides a model for integrative taxonomic revision in Octocorallia, combining molecular, morphological, and historical evidence. Recognition of Pseudothelogorgiidae fam. nov. resolves a long-standing taxonomic problem and firmly establishes this lineage as a distinct family within Malacalcyonacea. This taxonomic clarification provides a framework for future studies on related Indian Ocean octocoral lineages.

## Supplementary Material

XML Treatment for Pseudothelogorgiidae

XML Treatment for Pseudothelogorgia

XML Treatment for Pseudothelogorgia hartogi

## ﻿Additional information

### Use of AI

No use of AI was reported.

### Author ORCIDs

Kaveh Samimi-Namin https://orcid.org/0000-0002-7744-9944

Catherine S. McFadden https://orcid.org/0000-0002-8519-9762

### Data availability

All the data that support the findings of this study are available in the main text and supplementary material. Sequences included in the phylogenomic analysis are available in GenBank as BioProject #PRJNA1043120.

## Supplementary materials

Supplementary material 1 Sequence data included in phylogenomic analysis of *Pseudothelogorgia* Data type docx Explanation note Sequence data included in phylogenomic analysis of *Pseudothelogorgia*. Museum abbreviations: CAS, California Academy of Sciences; MZUCR, Museo de Zoología, Universidad de Costa Rica; NIWA, National Institute of Water and Atmospheric Research, NZ; RMNH, Naturalis Biodiversity Center (formerly Rijksmuseum von Natuurlijke Historie, Leiden); SBMNH, Santa Barbara Museum of Natural History; UF, Florida Natural History Museum; USNM, Smithsonian National Museum of Natural History. https://binary.pensoft.net/file/1479859This dataset is made available under the Open Database License (http://opendatacommons.org/licenses/odbl/1.0/). The Open Database License (ODbL) is a license agreement intended to allow users to freely share, modify, and use this Dataset while maintaining this same freedom for others, provided that the original source and author(s) are credited.Kaveh Samimi-Namin, Catherine S. McFadden
