# Supplementary material for: ﻿Integrative phylogenomic and morphological evidence for Pseudothelogorgiidae fam. nov., with a redescription of Pseudothelogorgia hartogi (Octocorallia, Malacalcyonacea)
Source: Zookeys. 2025 Nov 28;1261:201–21. doi: 10.3897/zookeys.1261.171874 (PMC12680942; doi:10.3897/zookeys.1261.171874)
Supplement: Supplementary material 1 — Sequence data included in phylogenomic analysis of Pseudothelogorgia [file zookeys-1261-201_article-171874__-s001.docx]

**Supplementary Table S1:** Sequence data included in phylogenomic analysis of *Pseudothelogorgia*.

Museum name abbreviations: CAS, California Academy of Sciences; MZUCR, Museo de Zoología, Universidad de Costa Rica; NIWA, National Institute of Water and Atmospheric Research, NZ; RMNH, Naturalis Biodiversity Center (formerly Rijksmuseum van Natuurlijke Historie, Leiden); SBMNH, Santa Barbara Museum of Natural History; UF, Florida Natural History Museum; USNM, Smithsonian National Museum of Natural History.

| **Family** | **Genus/Species** | **Specimen** | **GenBank SRA** |
| --- | --- | --- | --- |
| Astrogorgiidae | *Astrogorgia* sp. | RMNH.COEL.40875 | SRR10443770 |
| Discophytidae | *Discophyton rudyi* | SBMNH 145136 | SRR10443697 |
| Pseudothelogorgiidae fam. nov. | *Pseudothelogorgia hartogi* | UF 16054 | PRJNA1043120 |
| Isididae | *Isis hippuris* | CAS IZ196605 | SRR10443802 |
| Isididae | *Rumphella* sp. | RMNH.COEL.40991 | SRR10443780 |
| Keroeididae | *Keroeides* sp. | CAS IZ196833 | SRR10443824 |
| Plexauridae | *Eunicea calyculata* | USNM 1606825 | SRS19645580 |
| Plexauridae | *Eunicea clavigera* | USNM 1606821 | SRS19645514 |
| Plexauridae | *Eunicea flexuosa* | USNM 1606843 | SRS19645568 |
| Plexauridae | *Eunicea fusca* | USNM 1606831 | SRS19645587 |
| Plexauridae | *Eunicea fusca* | USNM 1606834 | SRS19645590 |
| Plexauridae | *Eunicea knighti* | USNM 1606828 | SRS19645584 |
| Plexauridae | *Eunicea laciniata* | USNM 1606837 | SRS19645594 |
| Plexauridae | *Eunicea mammosa* | USNM 1606844 | SRS19645569 |
| Plexauridae | *Eunicea succinea* | USNM 1606817 | SRS19645509 |
| Plexauridae | *Eunicea tayrona* | USNM 1606820 | SRS19645513 |
| Plexauridae | *Eunicea tourneforti* | USNM 1606826 | SRS19645582 |
| Plexauridae | *Eunicea tourneforti_*f*._atra* | USNM 1606824 | SRS19645515 |
| Plexauridae | *Muricea atlantica* | USNM 1606839 | SRS19645596 |
| Plexauridae | *Muricea atlantica* | RMNH.COEL.40834 | SRS12478882 |
| Plexauridae | *Muricea elongata* | USNM 1606823 | SRS19645512 |
| Plexauridae | *Muricea laxa* | USNM 1606842 | SRS19645567 |
| Plexauridae | *Muricea muricata* | USNM 1606840 | SRS19645565 |
| Plexauridae | *Muricea pendula* | USNM 1660639 | SAMN27177485 |
| Plexauridae | *Plexaura homomalla* | USNM 1606841 | SRS19645566 |
| Plexauridae | *Plexaura kuekenthali* | USNM 1606847 | SRS19645574 |
| Plexauridae | *Plexaura kuna* | RMNH.COEL.40836 | SRS2598938 |
| Plexauridae | *Plexaura kuna* | USNM 1606835 | SRS19645592 |
| Plexauridae | *Pseudoplexaura flagellosa* | USNM 1606815 | SRS19645507 |
| Plexauridae | *Pseudoplexaura porosa* | USNM 1606832 | SRS19645588 |
| Plexauridae | *Pseudoplexaura wagenaari* | USNM 1606845 | SRS19645571 |
| Plexauridae | *Swiftia exserta* | USNM 1660638 | SAMN27177484 |
| Pterogorgiidae | *Aliena parva* | MZUCR 3679 | PRJNA1043120 |
| Pterogorgiidae | *Dacrygorgia modesta* | USNM 1606549 | SAMN27177417 |
| Pterogorgiidae | *Muriceopsis flavida* | RMNH.COEL.40830 | SRR10443751 |
| Pterogorgiidae | *Pinnigorgia flava* | RMNH.COEL.40990 | SAMN27177444 |
| Pterogorgiidae | *Pterogorgia anceps* | RMNH.COEL.40837 | SRS19645575 |
| Pterogorgiidae | *Pterogorgia citrina* | USNM 1606829 | SRS19645585 |
| Pterogorgiidae | *Pterogorgia guadalupensis* | USNM 1606836 | SRS19645593 |
| Pterogorgiidae | *Stenogorgia casta* | USNM 1606518 | SAMN27177418 |
| Pterogorgiidae | *Stenogorgia casta* | RB-19-206 | SRS19645619 |
| Taiaroidae | *Taiaroa tauhou* | NIWA 28679 | SRR10443792 |
